# Cost-effectiveness Analysis of Rivaroxaban versus Acenocoumarol in the Prevention of Stroke in Patients with Non-valvular Atrial Fibrillation in Spain

**DOI:** 10.36469/9823

**Published:** 2016-02-01

**Authors:** Carlos Rubio-Terrés, Ruth Graefenhain de Codes, Darío Rubio-Rodríguez, Thomas Evers, Santiago Grau Cerrato

**Affiliations:** 1 Health Value, Madrid, Spain; 2 Bayer HealthCare, Barcelona, Spain; 3 Bayer Pharma AG, Wuppertal, Germany; 4 Hospital del Mar, Barcelona, Spain

**Keywords:** cost-effectiveness, acenocoumarol, rivaroxaban, non-valvular atrial fibrillation

## Abstract

**Objective:** The aim of this study was to evaluate, from the Spanish National Health System perspective, the cost-effectiveness of rivaroxaban (20 mg/day) versus use of acenocoumarol (5 mg/day) for the treatment of patients with non-valvular atrial fibrillation (NVAF) at moderate to high risk for stroke.

**Methods:** A Markov model was designed and populated with local cost estimates, efficacy and safety of rivaroxaban in stroke prevention in NVAF compared with adjusted-dose warfarin clinical results from the pivotal phase III ROCKET AF trial and utility values obtained from the literature. Warfarin and acenocoumarol were assumed to have therapeutic equivalence.

**Results:** Rivaroxaban treatment was associated with fewer ischemic strokes and systemic embolisms (0.289 vs. 0.300 events), intracranial bleeds (0.051 vs. 0.067), and myocardial infarctions (0.088 vs. 0.102) per patient compared with acenocoumarol. Over a lifetime time horizon, rivaroxaban led to a reduction of 0.041 life-threatening events per patient, and increases of 0.103 life-years and 0.155 quality-adjusted lifeyears (QALYs) versus acenocoumarol treatment. This resulted in an incremental cost-effectiveness ratio of €7045 per QALY and €10 602 per life-year gained. Sensitivity analysis indicated that these results were robust and that rivaroxaban is cost-effective compared with acenocoumarol in 89.4% of cases should a willingness-to-pay threshold of €30 000/QALY gained be considered.

**Conclusions:** The present analysis suggests that rivaroxaban is a cost-effective alternative to acenocoumarol therapy for the prevention of stroke and systemic embolisms in patients with NVAF in the Spanish healthcare setting.

## BACKGROUND

Atrial fibrillation (AF) is a cardiac arrhythmia associated with ageing, high blood pressure, valvular heart disease and other heart diseases.[1] Non-valvular AF (NVAF), the most common form of AF in developed countries, refers to cases in which the heart rhythm disorder occurs in the absence of rheumatic mitral valve disease, of an artificial heart valve, or of repair of the mitral valve. AF is associated with an increased risk of death (double that of without AF), cerebrovascular disease (5 times higher) and systemic embolism.[1] The prevalence of AF in the Spanish general population is estimated at 2%,[1] with 4.4% in the population older than or equal to 40 years of age,[2] reaching 10.9% and 11.1% in individuals over 60 and 79 years of age, respectively.[3,4]

The average annual cost of a patient with AF in Spain is estimated at €2365, €1008 of which correspond to hospitalisations, €723 to surgical interventions and €247 to the loss of labour productivity.[5] The cost of one of the complications of AF, cardioembolic stroke, has been estimated in the first 38 days of evolution at €13 353.[6] It is calculated that direct non-health costs of stroke, due to informal care of patients with sequelae, vary according to severity between €252 and €1031 in the acute phase (2 weeks) and between €1367 and €1942 per month in subsequent monitoring of the patient.[7]

Vitamin K antagonists (acenocoumarol and warfarin; VKA) are currently the standard treatment for stroke prevention in patients with AF.[8] In Spain, it is estimated that oral anticoagulation is used in 84% of patients with AF,[9] with 66% treated with a VKA in monotherapy.[10] However, the use of VKA is limited by the risk of bleeds, its narrow therapeutic margin and the drawbacks for the patient derived from the need for monitoring and the drug and food interactions. Although VKA, adjusting their doses, reduce the risk of stroke by 64% versus placebo, with warfarin the risk of suffering additional and intracranial bleeds is doubled.[11] In Spain, oral anticoagulant therapy is mostly conducted with acenocoumarol, while warfarin is used in English-speaking countries.[12] According to the available data, it is assumed that both drugs have a similar effectiveness in clinical practice.[12,13]

As recommended by the European Society of Cardiology,[14] new direct oral anticoagulants (DOACs), like rivaroxaban, are preferable to the VKA to treat most cases of NVAF, as they are not inferior regarding effectiveness and they reduce the number of intracranial bleeds.

Recently, DOACs such as rivaroxaban (once daily), dabigatran etexilate (twice daily), apixaban (twice daily) have been approved as possible alternatives to VKA therapy. Rivaroxaban (Xarelto®) is a highly selective, oral, once daily, direct factor Xa inhibitor that has shown a favourable risk-benefit profile compared with warfarin in the prevention of stroke and systemic embolism events. In the phase III study (ROCKET AF), rivaroxaban demonstrated a 21% risk reduction in event rate for stroke and systemic embolism (hazard ratio [HR]: 0.79; 95% confidence interval [CI]: 0.66–0.96; $p<0.001$ for non-inferiority) while on-treatment compared with warfarin, and a significant reduction in the most serious complications of warfarin therapy, i.e. intracranial haemorrhage and fatal bleeding.[15,16]

The differences observed between rivaroxaban and acenocoumarol/warfarin, could have a health and economic impact, and therefore an analysis of the cost-effectiveness of stroke prevention with rivaroxaban or acenocoumarol in patients with NVAF was performed.

## METHODS

### Economic Model

To analyse the cost-effectiveness of stroke prevention with rivaroxaban or acenocoumarol in patients with NVAF, a Markov model was used with the structure shown in Figure 1 and the methodology and results of which have been published previously for Belgium,[15] Portugal,[17] and Greece.[18] A hypothetical cohort of 1000 patients with an average age of 73 years (median age in ROCKET AF trial)16 and NVAF at moderate 1000 patients with an average age of 73 years (median age in ROCKET AF trial)[16] and NVAF at moderate ($CHADS_2$ score =2) to high risk ($CHADS_2$ score ≥3), enters the model and receives chronic treatment with rivaroxaban (20 mg daily) or an adjusted dose of acenocoumarol with a target INR of 2.5 (in the analysis a dose of 5 mg daily was considered, the daily dose recommended by the World Health Organization).[19] In both cases, the patients were treated with aspirin (ASA) in the event of discontinuing the initial treatment.

The clinical data obtained in the phase III ROCKET AF clinical trial[16] and the Spanish health osts were entered in the model, with the aim of calculating the incremental cost-effectiveness rate (ICER) of rivaroxaban compared to acenocoumarol.

A Markov model simulates the evolution in time of the disease of a theoretical cohort of patients. The entire cohort is initially in a given state of health. Later, some of the patients in the cohort are moved from one state to another with a given probability called "transition probability". These transition probabilities are calculated from clinical studies conducted with real patients.[20]

The patients enter this model with stabilised NVAF and receive the anticoagulant therapy (rivaroxaban or acenocoumarol). Then, every 3 months (called "cycles" of the model) the patients may remain stable (without experiencing events) or may suffer from one of the following events (Figure 1): major or minor ischemic stroke (IS), systemic embolism (SE), myocardial infarction (MI), major or minor extracranial bleed, intracranial bleed (IC) or death due to NVAF or other causes.[18] In the case of suffering from any of these complications, that patient would be assigned the costs and the loss of utilities (quality of life) corresponding to the acute episode. The major complications were classified as transient (non-boxed states in Figure 1) or permanent effects (boxed in Figure 1). Bleeding events were categorised as major extracranial, clinically relevant non-major (CRNM) extracranial, and intracranial as defined in the ROCKET AF study.[15,16]

The probability of the patients having a given event is determined by the event rate observed in the ROCKET AF clinical trial. Patients experiencing an ischemic stroke were presumed to continue with their anticoagulant therapy if they were already on therapy or to re-initiate it if they had discontinued. The model considered that the patients could not experience two acute events in the same cycle.

The modified Rankin Scores (mRS) used in the ROCKET AF trial to categorise strokes as either minor (mRS score 0-2) or major (mRS score 3-5) were considered.

The time horizon was 30 years, with the aim of finalising the model simulation with the death of the entire hypothetical cohort of patients.

**Figure 1.** Overall Model Structure

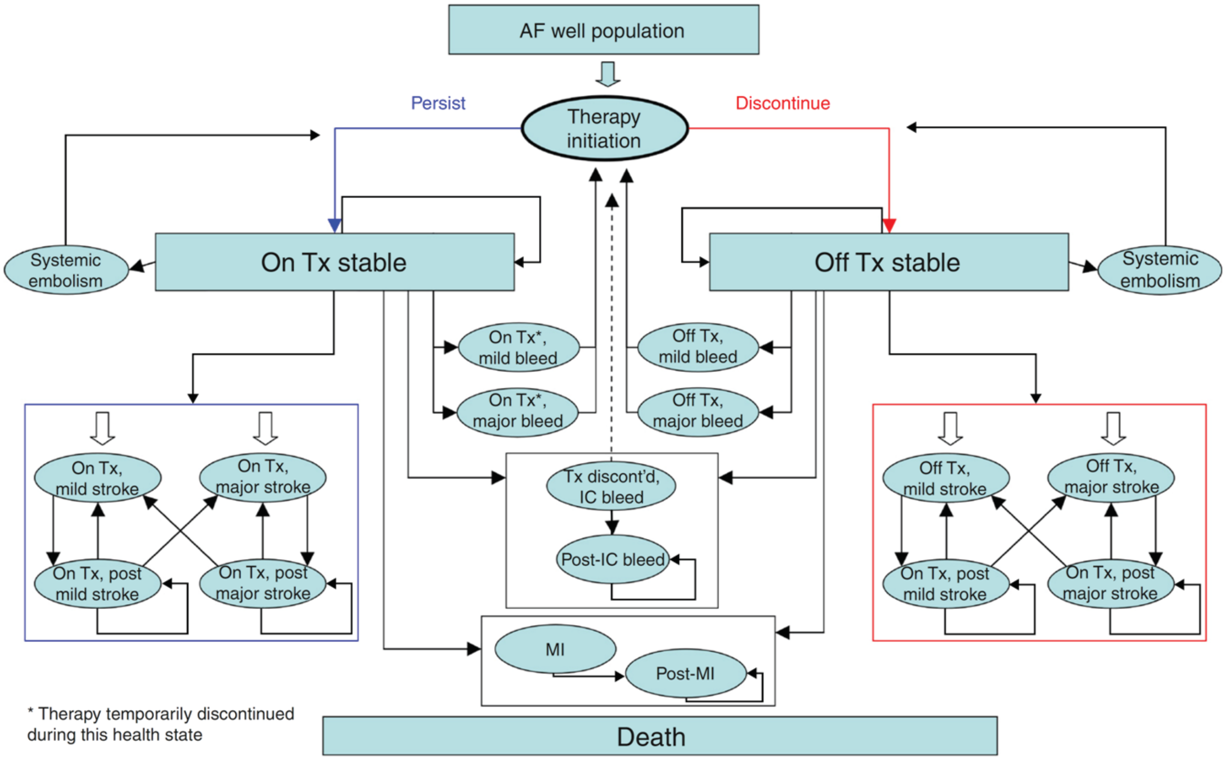

AF: atrial fibrillation; IC: intracranial; MI: myocardial infarction; Tx: therapy

## Efficacy Data

The main clinical efficacy and safety data (transition probabilities) introduced in the model come from the ROCKET AF clinical trial[16,21] (Table 1) as it is the only study that has compared rivaroxaban and warfarin head-to-head (comparable to acenocoumarol).

**Table 1.** Overview Clinical Input Data (95% CI)

| Event (source) | Baseline Risk (%) (3 months) | RR Rivaroxaban vs. Acenocoumarol | References |
|---|---|---|---|
| Ischemic stroke | 0.36 (0.27-0.45%) | 0.94 (0,75-1.17) | 16 |
| Of which major | 52.5 (47.6-57.3%) | - | 21 |
| Of which minor | 47.5 (42.7-52.4%) | - | - |
| Systemic embolism | 0.05 (0.00-0.76%) | 0.23 (0.09-0.61) | - |
| Extracranial CRNM bleed | 2.97 (2.81-3.15%) | 1.04 (0.96-1.13) | 16 |
| Extracranial major bleed | 0.69 (0.60-0.75%) | 1.14 (0.98-1.33) | 16 |
| Intracranial bleed | 0.19 (0.03-1.04%) | 0.67 (0.47-0.93) | 16 |
| Myocardial infarction | 0.28 (0.23-0.33%) | 0.81 (0.63-1.06) | 16 |

CRNM: clinicallly relevant non-major; RR: relative risk

Baseline event rates were obtained from the patients treated with warfarin in the clinical trial and converted into quarterly rates with acenocoumarol and rivaroxaban in the model, as described by Briggs et al.[22] For example, the baseline risk of stroke was 1.42% per year with warfarin in the clinical trial which was converted into a quarterly rate of 0.36% for the model. For rivaroxaban, this rate was multiplied by a relative risk of 0.94, resulting in a quarterly event rate of 0.34%.[15,16] Event rates for systemic embolism, bleeds and myocardial infarction were calculated the same way. The baseline mortality of the Spanish population was obtained from the database of the National Institute of Statistics.[23] Specific mortalities of clinical events of the patient with NVAF included in the model are summarised in Table 2.[24-26] These were obtained mainly from the ROCKET AF trial,[24] except for the long-term mortality rate of major stroke[25] and for myocardial infarction[26] that were obtained from the literature.[15] It was assumed that no deaths would occur as a result of the acute phase and during follow-up of minor stroke, CRNM extracranial bleeding and systemic embolism. Discontinuation rates for treatment with rivaroxaban (8.90% in the initial and 4.39% in the subsequent cycles) and acenocoumarol (8.00% in the initial and 4.46% in the subsequent cycles) were obtained from the ROCKET AF study.[15,16]

**Table 2.** Mortality Rates (95 % CI)

| Health State (source) | Event-related Mortality Rate per 3-month cycle (%) | References |
|---|---|---|
| Major stroke | 12.6 (9.4–15.7%) | 24 |
| Post-major stroke | 2.63 (0.91–13.5%) | 24, 25 |
| Minor stroke | N/A* | - |
| Systemic embolism | N/A* | - |
| Major extracranial bleed | 1.55 (1.16–1.94%) | 24 |
| Intracranial bleed | 38.85 (29.14–48.56%) | 24 |
| Post-intracranial bleed | 2.63 (0.91–13.5%)† | 24 |
| Myocardial infarction | 9.69 (7.27–12.11%) | 24 |
| Post-myocardial infarction | 2.68 (0–6.75%) | 24, 26 |

N/A not applicable.

*It is assumed minor stroke and systemic embolism have a case-fatality of 0% and thus mortality rate is equal to that in the general population.

†Assumed identical to post-major stroke mortality rate.

## Cost of Drugs and Events

The analysis was conducted from the perspective of the National Health System (NHS) in Spain.

Three types of costs were considered: that of acquiring the anticoagulant drugs,[27] that of administration/ monitoring of them and that of the events in the patient with NVAF with each treatment (Table 3).[19,28,29] The drug costs (public prices) were applied with a reduction of 7.5% (rivaroxaban) or of 15% (acenocoumarol, acetylsalicylic acid) according to Spanish law. When patients are initiated on acenocoumarol therapy, it is recommended that they visit their physician regularly to make the dose adjustments required to maintain the target INR of 2.5 (2.0 to 3.0).[15,30] To do this, as in other previously published Spanish studies, it was assumed that the annual cost is about €320[30] and the unit cost of the monitoring visit is half the cost of the routine visit to the general practitioner (Table 3).[19,29] For acetylsalicylic acid and rivaroxaban the model considers that patients would visit their GP twice a year (range: 0 to 8 visits per year) as no monitoring is required for these therapies.[15] The costs of events of the NVAF were also obtained from Spanish sources.[19,28,29] All costs are presented in Euros (€) of 2015. An annual discount rate of 3.0% was applied for the health costs and effects according to current recommendations in Spain.[31]

**Table 3.** Drug, Monitoring, and Event Costs (€2015)

| Item | Base Case | Low - High | Reference/Comment |
|---|---|---|---|
| **Drug cost (daily)** | | | |
| Rivaroxaban | 2.69 | - | 27 |
| Acenocoumarol | 0.13 | - | 27 |
| Acetil salicilic acid | 0.04 | - | 27 |
| **Monitoring cost** | | | |
| Acenocoumarol monitoring visit | 17.66 | 14.13 - 21.19 | Spain public prices* |
| Primary care medical visit | 35.32 | 28.26 - 42.38 | Spain public prices* |
| **Event cost** | | | |
| Minor stroke (per event) | 5070 | 4776 - 7164 | 19 |
| Minor stroke follow-on care (per quarter) | 360 | 288 - 432 | 19 |
| Major stroke (per event) | 6951 | 5561 - 8,341 | 19 |
| Major stroke follow-on care (per quarter) | 6246 | 4997 - 7495 | 28 |
| Stroke rehabilitation (per day) | 95.50 | 72.14 - 119.89 | 29 |
| Systemic embolism (per event) | 2846 | 2277 - 3415 | 19 |
| CRNM extracanial bleed (per event) | 35.32 | 28.26 - 42.38 | Assuming 1 medical visit |
| Major extracanial bleed (per event) | 3432 | 2745 - 4118 | 19 |
| Intracranial bleed (per event) | 7515 | 6012 - 9018 | 19 |
| Intracranial bleed rehabilitation (per quarter) | 8595 | 6876 - 10 314 | 29 |
| Myocardial infarction (per event) | 4989 | 1074 - 5201 | DRG 121, 122 and 123* |

*Spanish region health care public prices. CRNM: clinicallly relevant non-major

## Health-related Outcomes

The differences in effectiveness of the therapies were measured in life-years gained (LYG) and qualityadjusted life-years (QALY) gained. The utilities of the different health states used to calculate QALYs were obtained from studies conducted in patients in Europe or the United States with atrial fibrillation (Table 4).[15,32-39]

**Table 4.** Utility Values

| Description | Mean | Range | Reference |
|---|---|---|---|
| Stable - not on therapy | 0.779 | 0.635-1 | 15, 32 |
| Stable - on therapy | 0.779 | 0.635-1 | 15, 32 |
| Utility decrement used for acenocumarol | 1.000 | 0.920-1 | 15, 37 |
| Minor stroke | 0.641 | 0.550-0.660 | 15, 37 |
| Post-minor stroke | 0.727 | 0.538-0.772 | 15, 34 |
| Major stroke | 0.189 | 0.142-0.236 | 15, 37 |
| Post-major stroke | 0.487 | 0.078-0.710 | 15, 34 |
| Systemic embolism | 0.679 | 0.660-0.692 | 15, 39 |
| Minor bleed | 0.796 | 0.794-0.789 | 15, 39 |
| Major bleed | 0.618 | 0.590-0.645 | 15, 39 |
| Intracranial bleed | 0.600 | 0.020-0.635 | 15, 35 |
| Post-intracranial bleed | 0.740 | 0.078-0.772 | 15, 33 |
| Myocardial infarction | 0.667 | 0.501-0.799 | 15, 36 |
| Post-myocardial infarction | 0.703 | 0.528-0.799 | 15, 38 |
| Death | 0.000 | 0 | N/A |

### Base-case and Sensitivity Analysis

A base-case was analysed, with the average values of all parameters. To check the stability of results simple univariate sensitivity analyses were conducted (in each sensitivity analysis the baseline value of a variable is modified applying its end values) for all the variables of the model, modifying them according to their CI 95% or, if not available, ±25% or, in the case of costs, ±20%, considering that these variations would include the most relevant uncertainty of model.[15] A probabilistic analysis (second order Monte Carlo simulation) was also performed to check the uncertainty of the model's variables. For this it was assumed that the baseline risks would be adjusted to the beta distributions, that the relative risks would be adjusted to log-normal distributions, costs and utilities to gamma and beta distributions, respectively. One thousand simulations were performed modifying all the variables of the model.[18]

## RESULTS

### Episodes Avoided

According to the results generated from the model applied to a cohort of 1000 patients with NVAF, with rivaroxaban a number of events would be avoided compared to acenocoumarol: 11 ischemic strokes and systemic embolisms, 16 intracranial bleeds and 14 myocardial infarctions (Table 5).

### Cost-effectiveness

Consequently, in each patient treated with rivaroxaban instead of acenocoumarol more years of life would be obtained (0.103 LYG) and more quality-adjusted life years (0.155 QALY gained) (Table 5). Moreover, the incremental costs per patient treated with rivaroxaban would be €1092 for the NHS. This difference in costs is mainly due to higher reduction in mortality obtained with rivaroxaban (0.103 life-years gained per patient versus acenocoumarol) and the consequent longer duration of treatment in patients treated with rivaroxaban and its higher acquisition cost. The cost per LYG was €10 602. The cost per QALY gained was €7045 (Table 5). These results indicate that, compared with acenocoumarol, rivaroxaban is cost-effective in the prevention of strokes in patients with NVAF in Spain, as the cost per LYG or QALY gained is well below the €30 000, which is the threshold generally accepted in Spain.[40]

**Table 5.** Cost-effectiveness Results and Events avoided over the Life-time of Patients

| Events per 1,000 patients | Rivaroxaban | Acenocoumarol | Difference |
|---|---|---|---|
| Ischemic strokes and systemic embolisms | 289 | 300 | -11 |
| Intracranial bleedings | 51 | 67 | -16 |
| Myocardial infarctions | 88 | 102 | -14 |
| **Cost and effectiveness results per patient** | | | |
| QALY | 7.767 | 7.612 | 0.155 |
| Life-years | 10.237 | 10.134 | 0.103 |
| Costs (€) | 28 100 | 27 008 | 1092 |
| Incremental cost-effectiveness ratio (€) | 7045 per QALY gained<br>10 602 per life-year gained | | |

QALY: quality-adjusted life year

Sensitivity Analysis

The sensitivity analyses confirmed that rivaroxaban is a cost-effective treatment versus acenocoumarol. The Tornado diagram (Figure 2) shows that all ICER were below the cost-effectiveness threshold. The variable with the highest ICER (€23 155) was the rate of discontinuation of treatment with rivaroxaban after the first cycle of 3 months.

According to the probabilistic sensitivity analysis, the probability that rivaroxaban is cost-effective versus acenocoumarol is 89.4% (Figures 3 and 4).

**Figure 2.** Tornado Diagram showing the Main Drivers (variables and sensitivity ranges) of the Incremental Cost-Effectiveness Ratio (ICER)

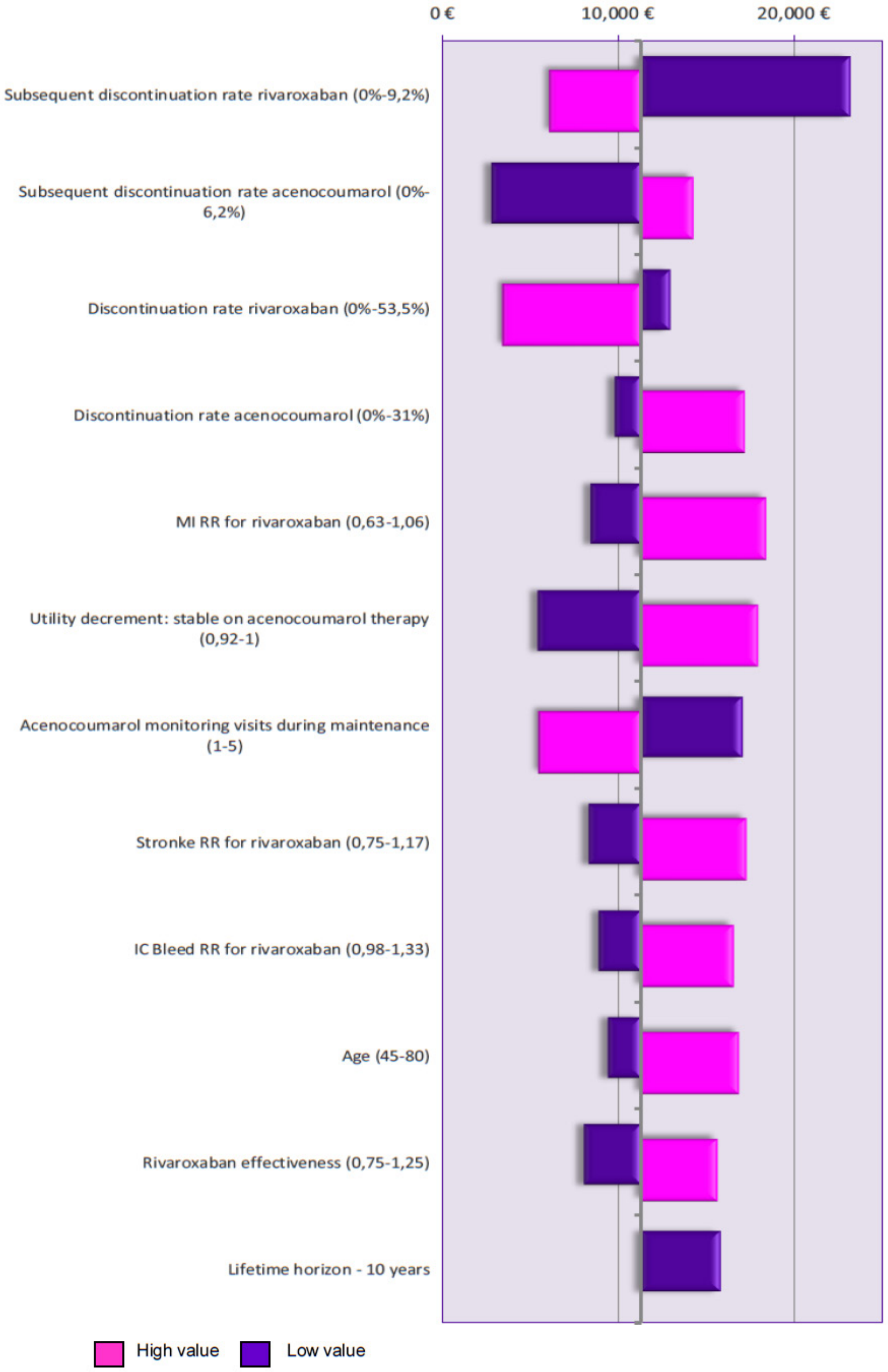

RR: relative risk

**Figure 3.** Probabilistic Sensitivity Analysis

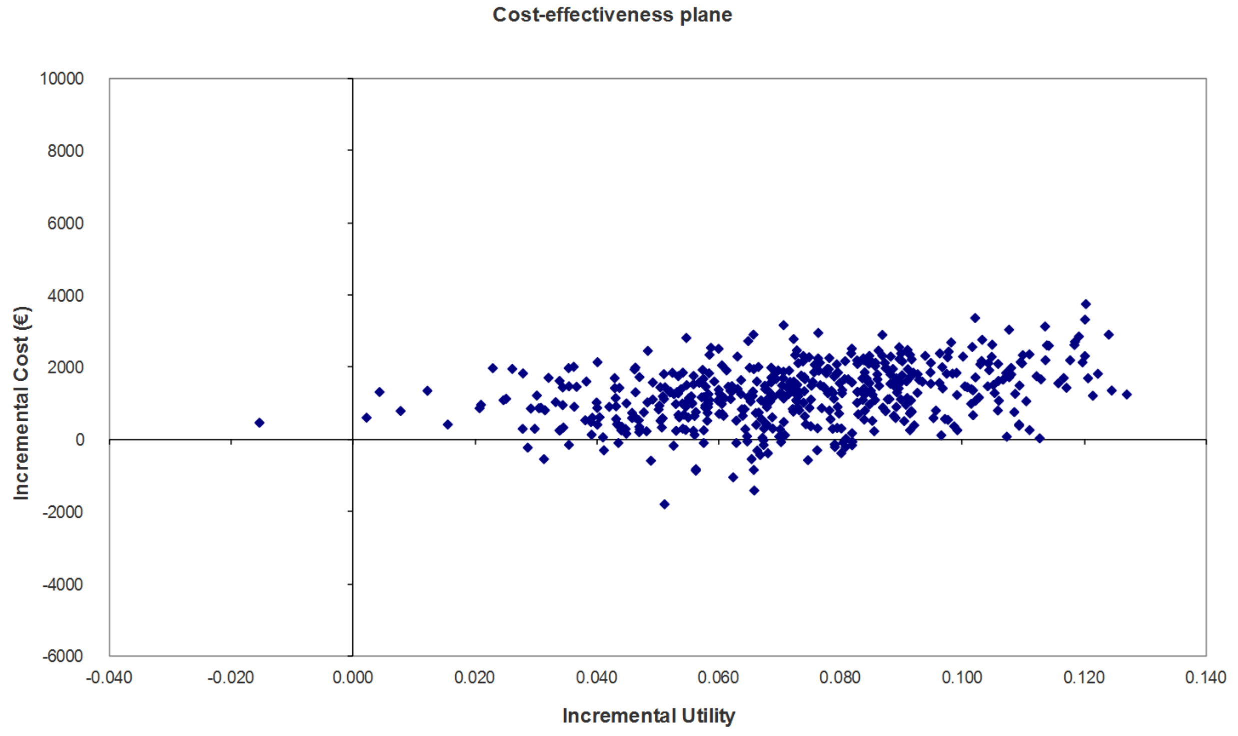

Cost-effectiveness plane. Sensitivity analysis indicated that these results were robust and that rivaroxaban is cost-effective compared with acenocoumarol in 89.4% of cases should a willingness-to-pay threshold of €30 000/QALY gained be considered.

**Figure 4.** Cost-effectiveness Acceptability Curve

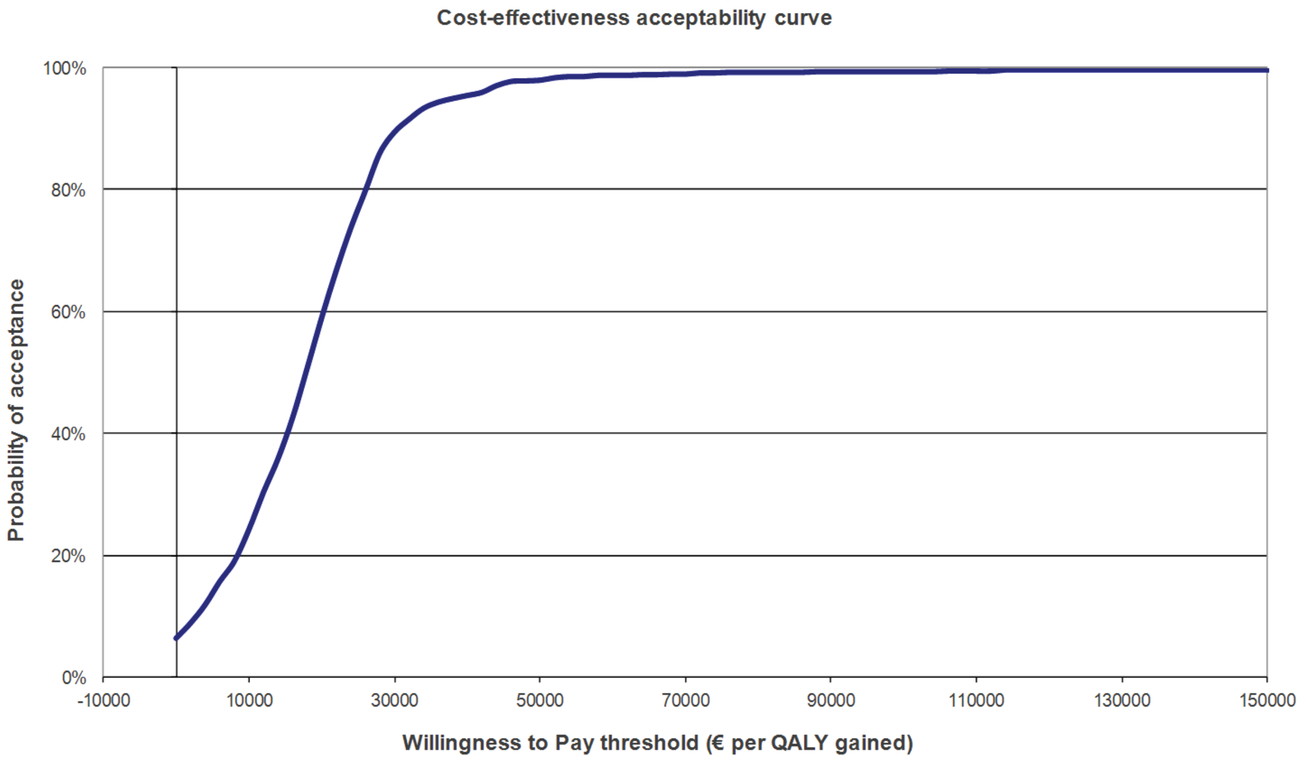

## DISCUSSION

Although acenocoumarol and warfarin are common treatments for NVAF, their use has limitations due to their narrow therapeutic margin, the risk of bleeds and the drug and food interactions.[41,42] Patients treated with VKA require frequent monitoring of the INR and of the subsequent dose adjustments. According to the meta-analysis by Baker,[43] patients treated with warfarin only remain 55% of the time in the INR range. In anticoagulated patients with cardioembolic stroke, 67.6% have a subtherapeutic INR at the time of stroke.[44]

Rivaroxaban is a new anticoagulant that statistically significantly reduces the combined risk of stroke and systemic embolism, and intracranial bleeds compared to VKA.[16] According to this model and the assumptions adopted, rivaroxaban can be a cost-effective treatment compared with acenocoumarol in preventing stroke in patients with NVAF in Spain.

### Limitations of the Model

In assessing these results we must take into account that this is a theoretical model that is, by definition, a simplified simulation of reality. Pharmacoeconomic models allow making economic simulations of complex drug-related health processes. They are especially useful when attempting to simulate the evolution of a disease beyond the duration of the clinical trials.[45] Markov models with Monte Carlo simulation are preferable to deterministic models when it comes to chronic diseases such as NVAF, as they simulate the evolution of the long-term disease better. The deterministic Markov model is based on the evolution of a hypothetical cohort of patients. However, the probabilistic analysis simulates the evolution of individual patients, based on values of the randomly taken variables, which is closer to clinical reality.[19] The efficacy and adverse effects data used in the model have been obtained from a randomised clinical trial conducted in 14 264 patients with NVAF,[16] which provides a high level of evidence in that regard.

Acenocoumarol is the most commonly used VKA in Spain.[46] One of the main assumptions of the model was the therapeutic equivalence of warfarin and acenocoumarol, so the values obtained in the ROCKET AF clinical trial versus warfarin[16] were assumed as effectiveness values of acenocoumarol. This assumption can be considered a limitation of the study, since although both drugs may have a similar effectiveness in clinical practice,[12] there is however an observational study in which no significant differences were observed in the quality of the INR control, but rather a greater duration of the time in therapeutic control in patients treated with acenocoumarol, versus those treated with warfarin (37.6% vs. 35.7%, P = 0.0002).[13] In another observational study conducted in Poland, it was concluded that switching acenocoumarol to warfarin in patients with unstable anticoagulation can improve anticoagulation control.[47] However, in other previously published Spanish economic studies it has been considered that acenocoumarol and warfarin are perfect substitutes, in both efficacy and safety, and in the use of the resources involved.[19,29,48] The costs assumed in the model for the acute phase of stroke and the bleeds were mostly obtained from one Spanish study.[19]

The cost of the drugs was calculated considering a dose of 5 mg/day of acenocoumarol, the Defined Daily Dose (DDD) of the World Health Organization. However, it should be noted that this assumption can only be used to calculate the cost of treatment with acenocoumarol, a variable that does not significantly affect the outcome because of the low price of the drug.[19]

The utilities of the model were obtained from several studies in Europe and the United States in patients with atrial fibrillation.[15,32-39] On the validity of these utility data regarding the Spanish population, in a study based on 83 000 assessments of 44 health states with EQ-5D, conducted in six European countries including Spain, there was greater variability between individuals than between countries.[49]

Despite the aforementioned limitations of the model, the results are consistent since the favourable outcome with rivaroxaban is maintained in both the deterministic sensitivity analyses and in the probabilistic analyses, with a high probability (89.4%) of rivaroxaban being cost-effective vs. acenocoumarol.

In 2012, the preliminary results of the adaptation of this model were presented in a congress, with similar conclusions, although without results of a probabilistic analysis.[29] Results for Belgium,[15] Portugal[17] and Greece[18] have been previously published using the same model. The study from Belgium concludes that, compared to acenocoumarol, rivaroxaban is cost-effective (for a threshold of €35 000/QALY) in 87% of cases.[15] The study for Portugal was made from the perspective of society, also including direct non-health costs (of rehabilitation), concluding that, compared to acenocoumarol, rivaroxaban is cost-effective (for a threshold of €30 000/QALY) in 72% of the simulations.[17] Finally, in the Greek study rivaroxaban would be cost-effective in 100% of cases.[18]

It is interesting to compare the results of this study with those of other Spanish studies on the costeffectiveness of apixaban and dabigatran vs. acenocoumarol in preventing stroke in patients with NVAF. In this regard, from the perspective of the NHS, the cost per QALY gained would be €12 825 with apixaban[19] and €17 581 with dabigatran These ICER are higher than those obtained with rivaroxaban (€7045) However, it should be noted that these results are not exactly comparable because the three models were different in structure, design and assumptions adopted; however they reflect the fact that the DOACs available in Spain are cost-effective options in the current Spanish health context.[19] In this regard, from the analysis of numerous meta-analyses have compared the effectiveness of these three DOACs[50-60] it can be concluded that although there are probably no significant differences between them, they significantly reduce the risk of stroke and death versus acenocoumarol.

According to the premises assumed in this model, it can be concluded that rivaroxaban can be with a high probability (89.4%, according to the probabilistic analysis) a cost-effective treatment compared to acenocoumarol in the prevention of stroke in patients with NVAF treatment, according to the costeffectiveness threshold generally accepted in Spain. The stability of the results obtained in the base-case of the analysis has been confirmed in deterministic and probabilistic sensitivity analyses.

## CONFLICT OF INTEREST DECLARATION

Study financed by Bayer HealthCare. Ruth Graefenhain de Codes works for Bayer HealthCare, Barcelona, Spain. Thomas Evers works for Bayer Pharma AG, Wuppertal, Germany. The other authors have received fees from Bayer HealthCare or Health Value for their collaboration in consultancy work.
